# Supplementary material for: Trichuris muris infection drives cell-intrinsic IL4R alpha independent colonic RELMα+ macrophages
Source: PLoS Pathog. 2021 Jul 30;17(7):e1009768. doi: 10.1371/journal.ppat.1009768 (PMC8357096; doi:10.1371/journal.ppat.1009768)
Supplement: Attachment — Submitted filename: Forman_response to reviewers comments.docx [file ppat.1009768.s010.docx]

Dear Dr Nair,

Thank you for your email with reference to our manuscript submitted to PLOS Pathogens entitled **Trichuris muris infection drives cell-intrinsic IL4R alpha independent large intestinal RELMα+ macrophages** containing the reports of three reviewers.

We are pleased to note all of the reviewers agree on the importance of this study, and that it will be of interest to the mucosal research community. In particular Reviewer 1 highlights the work addresses an important and relevant gap of knowledge. Reviewer 2 observed that the mice we have generated will be a useful tool of interest to other researchers and Reviewer 3 praises the use of relevant controls and views the data of interest to the field of mucosal immunology.

We are most grateful to you and the reviewers for the helpful comments, which we consider will improve the paper.

We are therefore sending as requested:

[1] A detailed list of our responses to the reviewers’ comments and a description of the changes we have made in the manuscript. These include new *in vivo* data exploring RELMα expression in peritoneal macrophages in the IL4Rαfl CXC3CR1Cre mouse.

[2] Two versions of the revised manuscript: one with tracked changes denoting where the text has been changed and a clean version (uploaded as the manuscript file).

**Responses to Reviewers**

Throughout this response letter, the comments of reviewers are in normal text and underlined and our responses are italicised, followed where appropriate by a section of indented non-underlined text highlighting in yellow changes we have made.

**Part II – Major Issues: Key Experiments Required for Acceptance**

Please use this section to detail the key new experiments or modifications of existing experiments that should be absolutely required to validate study conclusions.

Reviewer #1: Major comments:

1. The absence of IL4Ra expression in macrophages is not fully characterized in colonic macrophages. The results are in Fig 1c and d are however convincing as IL-4c treatment did not results in the detection of an increased proportion of RELMa+ macrophages. However, there is no characterization of the CX3CR1cre Il4ra-floxed mouse. As acknowledged by the authors, CX3CXR1 is not restricted to macrophages and other cell types in these mice will also have impaired IL4Ra expression. This should be addressed in the steady-state and during infection (MFI for IL4Ra, RT-qPCR for Il4ra expression, and potentially genotyping of Cre-mediated deletion efficiency of the targeted exons) in sorted populations of macrophages. The results section refers to Yona et al to state that CX3CR1 is expressed in all macrophage subsets in the intestine, but this study describes the macrophage populations in homeostasis, not during inflammation.

*We are disappointed that the reviewer does not feel we have fully characterised the deletion of the IL4Rα in colonic macrophages in the IL4Rαfl CXC3CR1Cre mouse for the following three reasons:*

i) *Importantly, as well as our phenotypic analysis we have substantiated the loss of the IL4Rα on macrophages with a functional analysis through treating the mice with IL4 complex (Fig 1), which we note the reviewer states looks convincing. Thus this data demonstrates that even following exposure to high levels of IL4 after treatment with IL4c we see no upregulation of RELMα in colonic macrophages in the Cre+ mice. We believe this to be a much more robust approach to characterizing the absence of the IL4Rα in colonic macrophages than can be achieved via phenotypic based assessments.*
ii) *Moreover, we have corroborated our findings in the IL4Rαfl CXC3CR1Cre mice with data from a totally independent model system utilising IL4Rα KO/wild type mixed bone marrow chimeras.*
iii) *We have already included within the manuscript analysis of IL4Rα expression in colonic tissue post infection of Cre+ and Cre- mice*

2. The overall RNAseq analysis should be better detailed, such as PCA analysis and other QC parameters. Also, the rationale to choose the CD206+/- populations for analysis is described but is hardly followed in the following sections of the manuscript. The heatmaps that are shown showing the 20 genes up- or down-regulated only provide a limited information about the data, and also hide the statistical significance of the observed fold changes. A Volcanoplot (log2FC vs P values) and/or MA plot (normalized counts vs log2 FC) would be informative about the quality of the data and significance. Then, the conclusion that CD206+ macrophages are anti-inflammatory based on the regulated gene might well be true, but performing gene set enrichment analyses would be a substantial added value to support the conclusions.

*We sorted our intestinal macrophages on the presence/absence of the cell surface marker CD206 as a good surrogate for RELMα expression given that RELMα is an intracellular molecule. The manuscript subsequently then focusses on the alternatively activated macrophage markers RELMα, (YM1 and PDL2).*

*We apologise for the lack of detail within the RNAseq analysis. We have fully revised the figure and have now included as requested the PCA analysis, QC parameters and volcano plot. We have therefore altered the accompanying results text which now reads:*

Cells were pooled from two mice, sorted using FACS and isolated into two subsets based on surface expression of the marker CD206, chosen based on good co-expression with intracellular RELMα post *Trichuris* infection (Supp Fig1a). Counts per gene demonstrated good quality RNA seq reads (Supp Fig1b) and principal component analysis (PCA) analyses of the two monocyte / macrophage subsets revealed a detectable influence of animal sample on the gene expression profile (22%) but this was significantly weaker than the difference attributed to expression of CD206 (58% of the sample variance; Supp Fig1c). In addition, *Mrc1,* the gene encoding CD206 and *Retnla* the gene encoding RELMα, were significantly upregulated in the CD206 positive population, validating the sorting strategy (Supp Fig 1d). Importantly, volcano plot analysis of the differential gene expression between the 2 macrophage populations revealed that the CD206^+^ macrophage profile associated with genes such as *Retnla, Mrc1, Ccl8, CD163, Ccl6,* and *Gas6* which have all been described to be upregulated in alternatively activated macrophages [1] (Supp Fig 1d). In addition, IPA analysis demonstrated an enrichment in our dataset of genes associated with the inflammatory response (p=6.69x10^-20), inflammation of the gastrointestinal tract (p=6.76x10^-23) and inflammation of the large intestine (p=4.54x10^-18).

3. The authors observed RELMa+ macrophages in CX3CR1creIl4rafloxed mice, as well as in WT:IL4Ra-/- chimeras. This is an interesting observation, although the increased RELMa expression after T. muris infection in macrophages does not seem to be very strong (between naïve and infected mice). However, the authors do not show the data of IL4Ra-/- mice, and whether RELMa (and YM1, CD206, PD-L2) is also up in these mice.

*Whilst we agree this would be an interesting control to analyse unfortunately this is not possible, as, given the absence of IL-4 signalling in all cell types, the global IL4Rα-/- mouse is unable to mount a Th2 immune response and is susceptible to T. muris infection. Th1 dominated T. muris-susceptible mice therefore do not show any increase in RELMα macrophages within the intestine; as we only see RELMα macrophages in resistant mice. This is why we used the mixed bone marrow chimera approach. Thus, the IL4Rα KO/WT mixed bone marrow chimaera mouse is able to expel the parasite and enables a dissection of the importance of the IL4R on macrophages in driving RELMα expression in the context of worm expulsion and equivalent parasite exposure. We have clarified this within the text:*

However, as the IL4Rα global deficient mice do not develop a Th2 immune response they are unable to expel Trichuris and, as susceptible mice, do not drive a significant increase in RELMα+ macrophages in the intestine post infection [6]. Thus, RELMα macrophages only accumulate in the intestine in the *T. muris* mouse model in resistant mice*.* Therefore, we employed a mixed bone marrow chimera approach.

In contrast to PD-L2, RELMa and YM1 are secreted proteins that could be well produced by other another cell type in large quantities in response to IL-4 and IL-13 (or not) and phagocytosed by intestinal macrophages. This phenomenon was found in alveolar macrophages after N. brasiliensis infection (Krljanac et al., 2019 – ref 14).
Before concluding that intestinal macrophages express RELMa at similar levels in both mouse strains, RNA expression should be determined specifically in these cells to determine the expression levels of the Retnla gene. Unfortunately, the immunostaining in colon tissue sections does not allow to draw a clear conclusion.

*We thank the referee for raising this. Krljanac et al 2019 suggest that a small proportion (10%) of alveolar macrophages may acquire cytosolic proteins e.g. RELMα (not secreted RELMα) from adjacent pneumocytes in the lung alveoli. We agree that the possibility that the RELMα protein we see in both the WT and the IL4Rα deficient macrophages is actually derived from another cell type that has been phagocytosed by macrophages should be commented upon. In our study of intestinal macrophages post whipworm infection, three lines of evidence presented within our manuscript suggest that the RELMα + macrophages we detect are not RELMα+ due to their uptake of RELMα+ apoptotic cells, namely:*

(i) *RNAseq analysis (Supp Fig1c) demonstrates that Retnla is highly expressed at the mRNA level in WT intestinal macrophages post infection.*
(ii) *Analysis of RELMα expression within the colonic tissue demonstrates that unlike in the lung environment where there is an abundant RELMα expression by the epithelial cells during infection the colonic intestinal epithelial cells do not express significant levels of RELMα (Fig 3g).*
(iii) *Although other infiltrating cells are capable of expressing RELMα, analysis of the CD45+ RELMα+ cells within the inflamed colon (d18 post infection) demonstrates that macrophages are the predominant population expressing RELMα (see below).*

*We have added a statement within the discussion as below:*

Further, given that recent work has suggested that not all RELMα detected in lung macrophages is actually macrophage derived [2], with a small proportion (10%) of alveolar macrophages able to acquire cytosolic proteins e.g. RELMα from adjacent pneumocytes in the lung alveoli, the RNAseq analysis (Supp Fig1c) is important in demonstrating that Retnla is highly expressed at the mRNA level in intestinal macrophages post *T. muris* infection.

4. The choice of CX3CR1Cre instead of LysMCre is explained in the results-discussion section, with the given explanation that Lyz2 is not sufficiently expressed in gut macrophages during Schistosoma mansoni infection (Vanella et al, 2014). However, gut macrophages were not addressed in this study and it would have been relevant to compare the CX3CR1cre IL4rafloxed mice with LysMCre IL4rafloxed.

*We apologise for the error in the manuscript and have amended the section to remove references to gut macrophages:*

The Cre driver was chosen as it is known to efficiently delete floxed alleles in all intestinal macrophages [24]. This is important, as historically, determining the function of genes in macrophages has been confounded by inefficacies in deletion of floxed genes when using the LysM Cre driver [26]. The inefficient deletion of genes by LysM Cre is observed in subpopulations of macrophages which express low levels of Lyz2. Therefore, whilst LysM Cre is able to efficiently delete IL4Rα under steady state conditions, during inflammation a large proportion of macrophages retain IL4Rα expression [26, 27]

*Given the focus of our research was to assess the importance of IL4Ra signalling in colonic macrophages in driving RELMα expression we chose a Cre driver where we had confidence that the deletion would be efficient. Given that the LysM Cre driver is haunted with reports of incomplete deletion in inflamed tissues we chose the CX3CR1Cre driver for our studies. Whilst interesting, we feel that a comparison of Cre drivers in gut tissue is therefore outside of the aims of our current work and does not alter our conclusions.*

Reviewer #2: In view of these findings I wonder if the authors have considered the possibility of T. muris secretions/extracts inducing RELM-alpha on macrophages independently of Il-4R-alpha signalling.

*We agree with the reviewer that this an interesting possibility. We have now performed some additional studies using peritoneal macrophages (see below) and bone marrow macrophages (data not shown) stimulated with either T. muris excretions/secretions (ES) or IL4 as a positive controls. These experiments have demonstrated that T. muris ES is unable to stimulate production of RELMα in either IL4R α fl CX3CR1 Cre- or Cre+ mice. As anticipated, IL4 is able to stimulate the macrophages to produce RELMα in the IL4Rfl CX3CR1 Cre- but not the Cre+ mice. Thus we conclude that the presence of RELMa in the IL4Rfl CX3CR1 Cre+ mice is unlikely to be due to exposure to parasite ES.*

Reviewer #3: 1. The authors’ main conclusion in this manuscript is that large intestinal macrophages can adopt an alternatively activated phenotype characterized by expression of CD206, RELMa and YM1 independently of IL4Ra signaling. However, their experimental approach leaves alternative interpretations on the table – the phenotype could be tissue specific or helminth specific. Can the authors assess whether Trichuris muris infection induces IL4Ra-dependent mac polarization in other tissues, like the peritoneal cavity (or others, as physiologically appropriate)? This experiment will test whether large intestinal cells are uniquely IL-4Ra-independent in this infection setting and would add important information to the field and provide data to support the comparisons to H polygyrus and Litomosoides infections in text associated with Figure 4. An additional comparison that could add value to the field would be evaluating the IL-4Ra-dependence of RELMa+ intestinal macrophages in response to a helminth that has previously been shown to require IL4Ra for alternative activation of macrophages in another tissue (if feasible).

*We agree with the reviewer that assessing whether Trichuris muris infection induces IL4Rα -dependent macrophage polarization in other tissues, such as the peritoneal cavity is an important additional piece of data. Therefore we have included new in vivo data analysing the macrophage response in the peritoneal cavity during Trichuris infection. Trichuris infections do not drive a strong induction of RELMα expressing macrophages in the peritoneal cavity thus in order to maximise our opportunity to identify any RELMα+ macrophages in the peritoneal cavity in the absence of the IL4Rα we performed these experiments whilst treating mice in vivo with either an isotype control or anti-IFNγ antibodies to create a more strongly polarised Th2 environment. Our new data reveal that unlike the IL4Rα independence of RELMα expression in gut macrophages, peritoneal macrophages show a partial dependence on the IL4Ra. This data has now been incorporated into Figure 5 and is discussed in the text as follows:*

Further, to determine if the IL4Rα independence of RELMα expression is unique to colonic macrophages we analysed the expression of RELMα on peritoneal macrophages following *T. muris* infection. In order to create a strongly polarised Th2 environment we used the same approach as used in Figure 5a for intestinal macrophages and treated groups of infected mice with either an anti-IFNγ monoclonal antibody or an isotype control. In isotype treated animals there was no significant upregulation of RELMα expression in large peritoneal macrophages post infection (Fig 1e, Fig 5i); and no significant difference between the percentage of RELMα+ macrophages in IL4Rαfl/fl.CX3CR1Cre+ and IL4Rαfl/fl.CX3CR1Cre- mice (naïve IL4Rαfl/fl.CX3CR1Cre- 5.188±0.9823, IL4Rαfl/fl.CX3CR1Cre+ 4.477±1.632; infected isotype control treated IL4Rαfl/fl.CX3CR1Cre- 7.090±5.346 IL4Rαfl/fl.CX3CR1Cre+ 6.810±5.049; mean±SEM). However, following αIFNγ treatment, an increase in the percentage of macrophages expressing RELMα was observed in both the IL4Rαfl/fl.CX3CR1Cre- and IL4Rαfl/fl.CX3CR1Cre+ peritoneal macrophages but was significantly higher in the IL4Rαfl/fl.CX3CR1Cre- mice (Fig 5i,j). Therefore, whilst the expression of RELMα on colonic macrophages occurs independently of IL4Rα, expression of RELMα on large peritoneal macrophages is partially dependent on IL4Rα expression.

2. Data presented in Supplemental Figure 2a demonstrates that IL4Ra expression is reduced on colonic macrophages isolated from T muris-infected Cre+ mice compared to Cre- controls, but doesn’t conclusively show that it is absent on the Cre+ cells. Authors have access to IL4Ra-/- mice (as evidenced by data in Figure 4). A comparison of the IL4Ra MFI on colonic macrophages between the Cre+ cells and cells isolated from IL4Ra-/- would be much more informative and would be necessary to support the claim of efficient and sustained deletion in the Cre+ animals.

*The reviewer suggests that Supplemental Figure 2a doesn’t conclusively show that the IL4Rα is absent on the Cre+ cells. S/he suggests we include an additional negative control comparing the IL4Rα MFI on colonic macrophages between the Cre+ cells and cells isolated from IL4Rα-/- mice. We have not performed this control stain for the following three reasons:*

i) *Importantly, as well as our phenotypic analysis, we have substantiated the loss of the IL4Rα on CX3CR1+ cells with* ***a functional analysis*** *via treating the mice with IL4 complex (Fig 1), which reviewer 1 states looks convincing. Thus this data demonstrates that even following exposure to high levels of IL4 after treatment with IL4c we see no upregulation of RELMα in colonic macrophages in the Cre+ mice.*
ii) *Moreover, we have corroborated our findings in the IL4Rαfl CXC3CR1Cre mice with data from a totally independent model system utilising IL4Rα KO/wild type mixed bone marrow chimeras.*
iii) *We have already included within the manuscript analysis of IL4Rα expression in colonic tissue post infection of Cre+ and Cre- mice*

*Thus given our functional corroboration of the IL4Rα deletion on macrophages we feel it would be unethical to bring the IL4Rα KO mouse off its interrupted breeding cycle solely to use them as a staining control.*

**Part III – Minor Issues: Editorial and Data Presentation Modifications**
Please use this section for editorial suggestions as well as relatively minor modifications of existing data that would enhance clarity.

Reviewer #1: Minor comments:

- the use of the term ‘anti-inflammatory’ to characterize the macrophages present in the intestine during T. muris infection is probably not the best choice to describe the macrophage response in this context. There is an inflammation that develops against the presence of the worms in the large intestine, and although the inflammation is controlled and leads to repair mechanisms this is still ‘inflammation’. As the authors seem to consider important to use the correct terms with the description of M(IL-4) proposed in Murray et al. 2014; I would recommend to revisit their description of those ‘anti-inflammatory’ macrophages in the gut during worm infection.

*The reviewer raises a good point and we did in fact consider very carefully what term to use to best describe the macrophages present in the intestine during T. muris infection. On reflection we agree with the reviewer that ‘anti-inflammatory’ may not be the best descriptor and now refer to these macrophages throughout as alternatively activated.*

- there is no page number nor line numbers, which makes the manuscript difficult to read and review.
*We apologise for this admission and have added both line and page numbers.*

- the term ‘large intestinal macrophages’ could be confusing. The adjective ‘large’ refers to the intestine, but could also refer to ‘intestinal macrophages’; as there are ‘large macrophages in the intestine’.

*We agree and have changed our terminology to use ‘colonic’ instead of ‘large intestinal’ throughout the paper.*

- overall, the choice of using the terms ‘Cre+’ vs ‘Cre-‘ in the results section and in the figures; while also using the full name IL4Rafl/fl.CX3CR1Cre is confusing. Cre+ is to me too general and renders difficult the reading and understanding of the results without referring to the results section.

*We agree we could be clearer here and have adapted both the results text and figures to use the full name.*

- Results section 1 and FigS2: the gating strategies to analyze the selected macrophage population should be shown.

*We have added the gating strategies for these populations as supplementary figures 5-7.*

Moreover, the results section states that CD206, RELMa and Ym1 are down in the CX3CR1-Cre+ mice, but FigS2c, d and e show results from CD206, PD-L2 and RELMa. Which markers have been tested?

*We apologise for this inconsistency. We have checked the data and altered the results section where we had incorrectly stated Ym1 instead of PD-L2. The text now reads:*

Additionally, bone marrow macrophages from Cre+ mice were unable to upregulate the canonical anti-inflammatory macrophage markers CD206, RELMα and PD-L2 following exposure to IL4 (Supp Fig 2c-e).

- The results in FigS2a show MFI data for IL4Ra staining on gut macrophages at d35 post-infection, but panels b-e show bone-marrow macrophages. Even though there is a reduction in the MFI, this is not sufficient to assess the efficiency of IL4Ra impaired expression. Comparison with IL-4Ra-/- macrophages should be done, as well as a comparison between steady state and infected tissue (ideally including the main subpopulations of monocytes/macrophages – based on Ly6C and MHC-II expression (P1, P2, P3) and CD4/TIM-4)

*Whilst we agree that a comparison with the global knock out would be useful we have not performed this negative staining control for the following reasons:*

i) *Importantly, as well as our phenotypic analysis, we have substantiated the loss of the IL4Rα on CX3CR1+ cells with* ***a functional analysis*** *via treating the mice with IL4 complex (Fig 1), which the reviewer 1 states looks convincing. Thus this data demonstrates that even following exposure to high levels of IL4 after treatment with IL4c we see no upregulation of RELMα in colonic macrophages in the Cre+ mice.*
ii) *Moreover, we have corroborated our findings in the IL4Rαfl CXC3CR1Cre mice with data from a totally independent model system utilising IL4Rα KO/wild type mixed bone marrow chimera*
iii) *We have already included within the manuscript analysis of IL4Rα expression in colonic tissue post infection of Cre+ and Cre- mice compared to an FMO control*

*Thus given our functional corroboration of the IL4Rα deletion on macrophages we feel it would be unethical to bring the IL4Rα KO mouse off its interrupted breeding cycle solely to use them as a staining control.*

- In Figs. 1, S2, 3d-f, 4g-h,j, 5f-h: The entire population of macrophages seems to shift with antibody staining. Thus, an additional parameter to analyze are the median fluorescence intensities which should be shown instead of % of positive cells (or both information). In Fig. S2, MFI was chosen for IL4Ra but % were chosen in figs S2 c-e.

*We have now added in the MFI data for all the experiments as requested.*

- Fig. 3a: P1-P3 gates should be shown on the contour plots.

*We have added these labels to the contour plots to aid the reader.*

Moreover, the TIM-4 and CD4 staining should be shown as FACS plots as well.

*We have added in the Tim4 CD4 staining as representative FACS plots, see Sup Fig 3a*

In addition, the result section states that numbers of the macrophage subpopulations were affected upon infection, but the plotted data are percentages. Absolute cell numbers should be shown.

*We have shown the populations as percentages as in our hands, and as previously reported, during inflammation we can experience an artificially low yield of cells following gut digest. Therefore, we believe it is more accurate to report percentages rather than absolute cell numbers in these circumstances [3, 4]. We have altered the use of the word “numbers” in the results text so as not to be misleading.*

we observed a significant increase in the recruitment of monocytes into the intestine with an increase in the percentage of macrophages residing within the P1 and P2 gates

3.	Little MC, Bell LV, Cliffe LJ, Else KJ. The characterization of intraepithelial lymphocytes, lamina propria leukocytes, and isolated lymphoid follicles in the large intestine of mice infected with the intestinal nematode parasite Trichuris muris. J Immunol. 2005;175(10):6713-22. Epub 2005/11/08. doi: 10.4049/jimmunol.175.10.6713. PubMed PMID: 16272327.

4.	Little MC, Hurst RJ, Else KJ. Dynamic changes in macrophage activation and proliferation during the development and resolution of intestinal inflammation. J Immunol. 2014;193(9):4684-95. Epub 2014/09/28. doi: 10.4049/jimmunol.1400502. PubMed PMID: 25261482; PubMed Central PMCID: PMCPMC4201944.

- results section: “contribution of both CD45.1 and CD45.2 cells to CD4+ and CD8+ T cell derived IFNγ and IL13 production” referring to figS3c-e is confusing to conclude that IL4Ra-/- cells produce amount of the cytokines similar to the WT CD45.1 compartment. The proportion of IFNG+ of IL-13+ CD4 or CD8 T cells in both CD45.1 and CD45.2 should be shown for clarity.

*We apologise for the lack of clarity in this section. For completeness we have overlaid the contribution of CD45.1 and CD45.2 onto the figure (Supp Fig 4e) and we have clarified our text explaining the data to:*

Re-stimulation of MLNs with PMA and Ionomycin following infection demonstrated a significant induction of CD4 T cell-derived IFNγ and a trend for elevated CD4 T cell-derived IL13 in infected mice compared to naïve controls (Supp Fig 4c,d). In contrast, there was no upregulation of either IFNγ or IL13 derived from CD8+ T cells. Analysis of the contribution of CD45.1 and CD45.2 cells to both the whole CD4^+^ and CD8^+^ populations as well as the CD4^+^IFNγ^+^ and CD4^+^IL13^+^ populations revealed that the CD4+ cells were more likely to be from the CD45.2 (IL4Rα deficient) source. Therefore, despite lacking the IL4Rα, CD45.2 cells are able to produce both Th1 and Th2 cytokines (Supp Fig 4e).

Reviewer #2: The work is of interested and includes relevant essays with suitable controls.
The authors should show the data supporting expressing of intracellular RELM-alpha in CD206 macrophages post Trichuris infection. When analysing the gene expression of CD206 positive and negative macrophages (Supp Fig 1, a,b), the authors should stress that upregulation of Mrc1 in CD206 positive cells should be expected as this transcript encodes CD206. Indeed, this would be a nice validation of their sorting strategy.

*We thank the reviewer for their suggestion and have added the following sentence into the results text to incorporate this suggestion.*

In addition, *Mrc1,* the gene encoding CD206, was significantly upregulated in the CD206 positive population, validating the sorting strategy (Supp Fig 1d).

The authors demonstrate that different macrophage populations (peritoneal, colon, liver) from IL4R-alpha fl/fl.CXC3Cr1Cre+ mice fail to upregulate RELM-alpha in response to IL-4 complexes injected in the peritoneal cavity. I might have missed it but could not find the source and amount of Il-4C injected.

*We apologise for this omission and have added the following section into the methods text:*

***In vivo* IL4 complex treatment.** IL-4–anti–IL-4 mAb complex (IL4c) was generated by combining 5 µg of recombinant IL-4 (PeproTech) with 25 µg 11B11 (Bio X Cell) per mouse. Mice were treated intraperitoneally with IL4c and tissue harvested 48 hours later.

It is unclear what the authors refer to in legend of Figure 1 as (naïve; n=3-5, d18; n=6) as far as I can tell these animals were not infected

*We apologise for this error, the d18 mice were mislabelled and this should read IL4c, we have amended this and the figure legend now reads:*

Data are combined from two independent experiments (naïve; n=3-7, IL4c; n=6).

Also, I would like to enquire about their data presentation. All cells display some level of RELM-alpha expression based on the comparison of FMO control and specific labelling. Is this labelling real? If so it might be better to plot MFI rather than % of positive cells as at the moment it seems as most cells are positive and there is just upregulation of RELM-alpha expression.

*We have added in MFI data in addition to the % positive cells throughout for clarity.*

Data from bone marrow derived macrophages (Supp Figure 2 c-e) complement findings with ex vivo macrophages. These validation results would be more complete if in addition to % of cells expressing markers authors show MFI.

*We have added in the MFI data for the bone marrow derived macrophages (Supp Fig 2d,g,j)*

I was surprised by the relatively high expression of RELM alpha in unstimulated macrophages (15% of population already express it and only increases to 35% in response to IL-4) and I wonder if the authors would like to comment on that. In our experience CD206 is normally very well expressed by a high proportion of bone marrow derived. Based on the data less than 1-2% express CD206 and this % only increases to less than 10% in the presence of IL-4. Looking at their protocol, I did not notice any obvious reasons for these discrepancies. The authors included treatment with LPS and IFN-gamma, which as not been shown and collaction of sups that have not been characterised. Finally, I wonder if the Accutase treatment could account for the low levels of CD206 detected.

*The level of RELMα expression in naïve cells is not unusual in our hands and we are confident that our data is representative of levels seen in cells derived from mice in our CX3CR1creIL4R colony at Manchester. We agree there is a relatively low expression of CD206 on our bone marrow derived macrophages, although again we find this routinely in our hands. It is possible that this could be due to the accutase treatment, although we haven’t explicitly tested this. We have used accutase treatment rather than a physical scraping method to isolate cells as, in our hands, this results in a lower level of cell death. Importantly even with the low percentages, our data reveals that IL4 is only able to increase expression of CD206 in the bone marrow cells derived from the CX3CR1cre-IL4Rα mice.*

*Finally, we apologise for the mention of treatments and supernatant analysis we have not shown and have removed this from the methods.*

In figure 2 I would recommend including the label of the graphs in the actual figures as it is difficult to follow. Also, in my opinion it makes more sense to express Ab responses as titres rather than OD values.

*We are sorry that the figures were difficult to follow and have added additional labels as requested to the graphs. We have displayed the Ab responses as OD values as this is historically the standard for T. muris infection antibody data and thus allows comparison with the existing T. muris literature.*

In addition, it seems that there is a consistent increase in IL-4 and IL-13 production in mLNs cultures from infected animals and I question whether the authors would like to comment on this.

*We have commented on this within the text stating that we observe a mixed cytokine response in the draining lymph node with elevated levels of IFNγ, IL4 IL13 and TNF compared to naïve mice. This is what we would expect to see at day 18 post infection in a C57 Bl/6 mice where T. muris infection drives a mixed Th1/Th2 response and is well characterised in the literature*

Little MC, Hurst RJ, Else KJ. Dynamic changes in macrophage activation and proliferation during the development and resolution of intestinal inflammation. J Immunol. 2014;193(9):4684-95. Epub 2014/09/28. doi: 10.4049/jimmunol.1400502. PubMed PMID: 25261482; PubMed Central PMCID: PMCPMC4201944.

When showing the different macrophage populations during infection in Figure 3 the authors might want to include the P1, P2 and P3 labels in the dot-blots to guide the reader. I would also recommend showing MFI in addition to % positive cells.

*We thank the reviewer for their suggestion and have added both the labels and MFI analysis to Figure 3.*

In Figure 4 I confess that I found it difficult to follow the discussion regarding the migration and maturation of IL-4R alpha positive and negative monocytes during infection.

*We apologise to the reviewer for any difficulty found in following our discussion around figure 4. We have amended the section which now reads:*

Interestingly, and in contrast to our blood monocyte data where there was no alteration in chimerism, we saw a selection for IL4Rα-/- (CD45.2) monocytes in the colon, with a slight but significant decrease in cells derived from the CD45.1 (WT) compartment compared to in the bloodstream (Fig 4b,c).

Also, the PDL-2 labelling seems to indicate the presence of a small population of CD45+ (WT) macrophages expressing PDL2 cells so it would be important to show the gating strategy to calculate % of PDL-2 positive cells.

*We have added the gating strategy used into the supplementary figures (Supp Figure 8).*

Reviewer #3: 1. In the introduction, the authors state "This work is vital to inform strategies treating excessive, chronic intestinal inflammation as it highlights treatments which target the IL4R to promote anti-inflammatory macrophages are unlikely to be efficacious." This statement may not be entirely justified, as previous studies have demonstrated that IL-4 is sufficient to induce macrophage expression of RELMa, YM1, Arg1 and PDL2. Without a more broad characterization and accounting of functional differences between IL4-induced and Trichuris-induced but IL-4Ra-independent macrophages, authors are cautioned not to over-interpret the implications of their findings and to present them bearing in mind the difference between necessary and sufficient.

*We agree and have amended this section such that we do not over-interpret the implications of our findings. This section of the introduction now reads:*

This work is an important addition in our understanding of chronic intestinal inflammation and impacts upon strategies to treat these conditions. In particular, these findings highlight that targeting the IL4R to promote anti-inflammatory macrophages may not be efficacious.

2. In the introduction, authors state “Many laboratories have demonstrated that these markers are also IL4Ra-dependent in vivo and these have become reliable indicators of M(IL4), especially in the context of type 2 inflammation and helminth infections.” Please provide some references for readers who are interested in comparing the current findings with existing literature. Additionally, since the helminth family is quite broad and infections have their own unique features, it could be helpful to specify which helminth infections have been shown to induce IL4Ra-dependent macrophage polarization.

*We have added in a section to the introduction which references in more depth some of the work done on helminths and the induction of alternatively activated macrophages. The additional section reads:*

In particular, studies utilising the small intestinal nematodes *Heligmosomoides polygyrus* and *Nippostrongylus brasiliensis* have shown that RELMα^+^ Ym1^+^ CD206^+^ macrophages infiltrate the small intestine in an IL4/IL13 dependent manner [5, 6]. Similarly to these intestinal nematodes, it has been reported that the filarial nematode *Brugia malayi* drives an IL4Rα dependent RELMα^+^ Ym1^+^ Arg-1^+^ macrophage population within the peritoneal cavity [7].

3. Could not find Supplemental Figure Legends in the assembled PDF. Without those and other information, it’s not entirely clear what is being shown in Supp Fig 1. Can authors provide sort plots showing the sorted populations and/or include clarifying text: were macrophages and monocytes from P1, P2 and P3 included as a single population and sorted based solely on +/- expression of CD206? And was this only from T muris infected mice? What day post-infection? Finally, data shown in Supp Fig 1b (genes down-regulated in CD206+ vs CD206- mono/macs) is not mentioned in text.

*We apologise to the reviewer that they were unable to find the supplemental figure legends within the PDF. We have made several changes to Supp Fig 1 which has resulted in the removal of the previous Supp Fig 1b. In addition we have updated the legend which now reads:*

**S1 Fig. Confirmation of experimental models. Alternatively activated macrophages accumulate following T. muris infection.** Gut macrophages (gated as CD45+CD11b+CD11clow/intSiglecF-Ly6G-) were FACS-sorted from infected C57 BL6/J mice at d35 post infection based on expression of CD206 and RNA-Seq performed. (a) CD206 was chosen as a good cell surface surrogate for RELMα (b) quality control metrics, summary of reads counting. (c) PCA analysis of RNAseq data and (d) volcano plot analysis highlighting genes of interest.

4. Authors could consider annotating Fig 1 with source tissues (a, b: PEC; c, d: Colon; e, f: Liver). Similarly, in Fig 2b, annotating the days of infection would add clarity.
*We have added in these annotations to improve clarity.*

5. Text related to Supp Fig 2c-e indicates that CD206, RELMa and Ym1 were measured, but graphs shown quantify CD206, PDL2 and RELMa. Please revise for consistency. Additionally, can authors provide flow plots for transparency of what is being measured?

*We apologise for this inconsistency. We have checked the data and altered the results section where we had incorrectly stated Ym1 instead of PD-L2; it now reads:*

Additionally, bone marrow macrophages from IL4Rαfl/fl.CX3CR1Cre+ mice were unable to upregulate the canonical alternatively activated macrophage markers CD206, RELMα and PD-L2 following exposure to IL4 (Supp Fig 2c-e).

*For added transparency we have added the MFI data to accompany this analysis and representative flow plots.*

6. On Fig 3, can authors provide clarifying annotations eg P1, P2 and P3 on Fig 3a and a color-coded key for antigens stained for immunofluorescence (not IHC) images shown in Fig 3G.
*We have added both the annotations and colour coded key as requested.*

7. Is there a disconnect between the justification of using CD206 for sorting mono/macs for RNA Seq (“Cells were sorted using FACS and isolated into two subsets based on surface expression of the marker CD206, chosen based on good co-expression with intracellular RELMα post Trichuris infection (data not shown)”) and the data that is shown in Fig 3? At day 18 post-Trichuris infection, there is no significant increase in CD206-expressing colonic macrophages (~40-50% at d0 and d18), but RELMa+ colonic macrophages increase from ~25% in naïve animals to ~50% at day 18 post-infection. Can the authors provide some clarification? Perhaps showing flow plots of CD206 x RELMa would help.

*The preliminary data upon which we based our choice to use CD206 as an extracellular marker to enable our cell sorting was performed only at day 35 post infection (the time point the RNA seq was performed). In hindsight it is clear that there are some differences between these two M2 markers as we move from an uninfected to an infected state. Nevertheless, the purpose of this RNA seq experiment was just to show broadly how the macrophages within the intestine post infection fit within the defined translation landscape and thus we are confident in our data. We have added in a plot showing the co-expression of these two markers as Supp Fig 1a. In addition, the RNAseq data reveals that Retnla expression is clearly enhanced in the CD206+ population.*

8. Can authors add % +/- SEM of the IL4Ra-/- and WT populations on the flow plots of blood and large intestinal monocytes in Fig 4c?

*This data is quantified in Fig 4b, so we feel this is not necessary.*

9. RELMa staining does not appear to differ between naïve and infected mice in Fig 4i, despite quantification in Fig 4G indicating a 10-15% increase. Overlays, annotation of RELMa MFI or added gates for spatial context may be helpful.

*We apologise for the lack of clarity. We have added MFI data for this figure to help the visualisation of the data.*

10. In the Methods section, reference to E/S products can be removed.

*We actually use ES products in our MLN restimulations pertinent to Figure 2 and so feel it is appropriate to include this in the methods.*
